# Vaccine wastage in the Philippines: An examination of the contributory factors

**DOI:** 10.1002/puh2.50

**Published:** 2023-01-08

**Authors:** Gilbert D. Bernardino, Karolyn Rae A. Yogyog, Jude L. Tayaben, Paul Froilan U. Garma

**Affiliations:** ^1^ College of Nursing University of the Cordilleras Baguio City Philippines; ^2^ De La Salle Medical and Health Sciences Institute Dasmariñas Philippines; ^3^ College of Nursing Benguet State University, La Trinidad Philippines; ^4^ Institute of Health Sciences and Nursing Far Eastern University Manila Philippines

**Keywords:** COVID‐19, disinformation, health literacy, public–private partnerships, vaccine hesitancy, vaccine wastage

## Abstract

The global introduction of COVID‐19 vaccines provided relief to the burden inflicted by the SARS‐CoV‐2 virus. However, reports of vaccine wastage are still apparent in some countries, particularly in the Philippines. This paper examines the phenomenon of vaccine wastage with emphasis on factors related to improper storage and distribution and on vaccine hesitancy whose roots may be traced to a controversial vaccine rollout. The discussion elaborates on the following themes: (a) supply chain management, (b) limited health literacy, and (c) the unintended consequences of vaccine hesitancy. This paper asserts that addressing the problem of vaccine wastage may involve public‐private partnerships that are grounded in good governance and transparency. In addition, combatting disinformation may entail collaborative communication strategies that may tap into the valuable contributions of the health/natural sciences and the humanities.

## INTRODUCTION

Data from Johns Hopkins University [1] show that by October 2022, COVID‐19 cases in the Philippines have reached 3.98 million, while recorded deaths are at 63,477. The fight against the wide‐ranging effects of the virus was fortified with the global introduction of vaccines that aimed to increase the immunity of the general population. However, reports of vaccine wastage have made the goal of eradicating the virus even more challenging.

From February 28, 2021 through September 4, 2021, the Department of Health (DOH) in the Philippines reported a total of 4855 doses of vaccines that have been wasted [2]. The number of wasted vaccines represents about 0.01% of the total 28.8 million vials that have been delivered across all regions [2]. By August of 2022, a Senate Committee on Health hearing revealed that 20.7 million vaccines were wasted amounting to PhP 10.33 billion or more than USD 175 million [3]. Last May 2022, the DOH targeted to vaccinate 90 million Filipinos but was only able to vaccinate 72 million [3]. This paper explores the phenomenon of vaccine wastage in the Philippines. A report from the Asian Development Bank [2] claims that reasons for the wastage include (1) improper storage and distribution and (2) discrepancies from those listed in the target number of people to those who actually received the vaccines [2]. These factors will be explored in greater detail in the succeeding discussion of the limitations of the supply chain management in the Philippines alongside the occurrence of limited health literacy, and the tension‐ridden experience of the country on a controversial vaccine rollout.

## SUPPLY CHAIN MANAGEMENT

A rapid assessment and focus group discussion conducted by Nieva et al. [4] prior to the availability of COVID‐19 vaccines shows that some provinces still do not have specific policies and protocols in handling expired vaccines. While some provinces typically observe the first in first out (FIFO) system and monitor the inventory of every Rural Health Unit (RHU) on a monthly basis, this practice does not appear to be commonplace [4]. It was also found that prior to the rollout of COVID‐19 vaccines, only provincial health facilities were used to store vaccines that required a temperature range of –25°C up to 8°C [4]. It is noted that COVID‐19 vaccines can have different temperature requirements as some may need to be stored at temperatures that range from –60 to 90°C [5]. In addition, transporting COVID‐19 vaccines may take hours of travel in various terrains, and disruptions in power supply for the continuity of cold chain storage may potentially contribute to vaccine wastage [6]. Addressing the pandemic needs of people in far‐flung areas or those in geographically isolated and disadvantaged areas (GIDA) requires increasing the capacity and awareness of grassroots stakeholders to manage health problems.

## LIMITED HEALTH LITERACY

Health literacy, to some extent, is still considered to be a privilege of expert systems that dominate policy decision‐making tables. In 2020, it was reported that while there may be an oversupply of graduates in the field of information technology, there exists a shortage of science, technology, engineering, and mathematics graduates [6]. A global study in 2019, in addition, showed that the Philippines scored “significantly lower” for grade 4 assessments on science and mathematics, thereby lagging behind all 58 participating countries [7]. In a cross‐sectional survey conducted by Tolabing et al. [8], they found that Filipinos with limited health literacy have a national prevalence of 51.5%. With this in mind, there exists the possibility that some people still consume information presented in mainstream media even if they are not evidence based. With limited health literacy, people can be more likely to engage in risky behaviors and unhealthy lifestyles. These propensities, in the long run, can become contributory factors that can explain the occurrence of vaccine hesitancy and its unfortunate consequence, vaccine wastage.

## UNINTENDED CONSEQUENCES OF VACCINE HESITANCY

The problem of vaccine wastage can also be linked to the issue of vaccine hesitancy which has many roots in the Philippine society. The dengvaxia controversy that was intensified in 2017 for instance, unintentionally implanted a notion that vaccines are unsafe and can cause deaths [9]. Due to vaccine hesitation, an uptick in vaccine‐preventable diseases, particularly ones that had long been controlled such as measles, became apparent and only 60% of children were reported to have received their scheduled vaccines [10]. In the Philippines, poor working conditions and low remuneration continue to push health workers to more developed Western countries. Health literacy can be potentially enhanced and the problem of vaccine wastage may possibly be averted if the graduates of medical and allied health courses are largely retained and employed in grassroots and primary health facilities for the benefit of the populace. If local health workers are retained and deployed in community settings, the foundations of primary health care can be massively strengthened.

## ALTERNATIVE STRATEGIES

In this section, possible strategies that can be further enhanced to reduce the occurrence of vaccine wastage will be discussed. In particular, strengthening public–private sector partnerships and addressing the ills of disinformation will be elaborated.

The involvement of the private sector in the rollout of vaccines can help address some limitations in the level of supply chain management. In 2021, private sector companies were allowed to acquire vaccines provided that they participated in tripartite agreements with the Philippine national government [11]. The tripartite agreement specifies that the national government will shoulder the costs of adverse effects and that the vaccines must not be sold for profit [11]. With close collaboration with the private sector, the administration, distribution, and communication of the importance of the vaccine can become more efficient owing to the mobilization of drugs to various parts of the country. Despite the numerous benefits that can be derived from public–private partnerships (PPPs), PPPs can still be prone to corruption [12]. Private entities that wield significant power and are not closely supervised by government bodies in addition to the likelihood of bribery toward public officials are among the likely scenarios. As such, measures that aim to promote good governance and transparency must still be in place to ensure the efficiency of PPPs to reduce vaccine wastage.

Addressing the problem of vaccine hesitancy and wastage requires efforts that may be deemed unorthodox for some. Communicating health concepts and reminders, and engaging in respectful dialogues with other stakeholders need not always be the task of health experts when the inputs of people in the human and social sciences can be equally tapped in responding to the challenges of SARS‐CoV‐2 virus. While statistical messages convey essential empirical information, highlighting pro‐vaccine narratives drawn from the experiences of individuals may also be beneficial. This, in a way, captures the use of evidence‐based storytelling to help combat the emotional dimension of misinformation. Another way of reframing the situation, instead of merely labeling population sentiments as either pro or against vaccination initiatives, is to look into the spectrum of vaccine hesitancy as containing diverse socio‐cultural factors. Vaccine decisions, moreover, should take into account the different factors that affect an individual's decision‐making such as past experiences, family history, feelings of control, and conversations with friends. Given the socio‐cultural climate of the Philippines, these narratives have a level of impact on how people plan and execute their health‐related decisions.

People who accompany individuals in vaccination sites, even if they are not included in the masterlist, must be encouraged to receive the vaccine as well. Given the social cohesiveness of groups in the Philippine society, transdisciplinary efforts must be exerted at combatting disinformation to ensure a wider acceptance of the vaccine.

## CONCLUSION

With the continued emergence of COVID‐19 cases, efforts aimed at increasing the number of vaccinated individuals remain to be crucial. However, the number of wasted vaccines from problems related to supply chain management and vaccine hesitancy needs to be addressed at all fronts. While PPPs hold a key potential in improving the rollout of vaccines to various areas, its susceptibility to corruption requires critical planning and design. Furthermore, communicating the importance of the vaccine must not be the sole task of public health providers when the input of actors from the human and social sciences can be equally integrated.

## AUTHOR CONTRIBUTIONS

All authors participated in the collection and analysis of existing literature. All authors adequately participated in writing various versions of the paper. All authors have read and approved the final manuscript.

## CONFLICT OF INTEREST

The authors declare that they have no known competing financial interests or personal relationship that could have influenced the content of this manuscript.

## Data Availability

The data that support the findings of this study are available online from journal articles and news reports that have been specified in the list of references.

## References

[1] Johns Hopkins University . COVID‐19 data repository. October 16, 2022. Accessed October 16, 2022. https://github.com/CSSEGISandData/COVID‐19

[2] Asian Development Bank (ADB) . Vaccine needs assessment. Accessed October 16, 2022. https://www.adb.org/sites/default/files/linked‐documents/54171‐004‐ld‐01.pdf

[3] Senate of the Philippines . Hontiveros: Vaccine wastage doubled to P10B in August. August 15, 2022. Accessed October 16, 2022. https://legacy.senate.gov.ph/press_release/2022/0815_hontiveros1.asp

[4] Nieva RF Jr , Robles YR , Benosa CAC , Faraon ARG . Strengthening provincial supply chain management capacity for COVID‐19 vaccines. De La Salle University. Policy Brief, 2021;1(8). https://static1.squarespace.com/static/58ff0e1fa5790aa37e3fa35a/t/60ac71e2719b9c76b56bbca4/1621914130548/JRIG%2BPolicy%2BBrief%2BVolume%2B1%2BIssue%2B8.pdf

[5] CDC . Pfizer‐BioNTech COVID‐19 vaccine storage and handling summary. 2022. Accessed October 17, 2022. https://www.cdc.gov/vaccines/covid‐19/info‐by‐product/pfizer/downloads/storage‐summary.pdf

[6] Laforga BM . Philippines facing oversupply in IT graduates, STEM shortage. *Business World* . February 26, 2021. Accessed August 20, 2022. https://www.bworldonline.com/editors‐picks/2021/02/26/346960/philippines‐facing‐oversupply‐in‐it‐graduates‐stem‐shortage/

[7] Magsambol B . PH lowest among 58 countries in math, science – global assessment. *Rappler*. December 9, 2020. Accessed August 23, 2022. https://www.rappler.com/nation/filipino‐students‐lagging‐behind‐math‐science‐timms‐international‐results‐2019/

[8] Tolabing MCC , Co KCD , Mendoza OM , et al. Prevalence of limited health literacy in the Philippines: first national survey. Health Lit Res Pract. 2022;6(2):e104‐e112. 10.3928/24748307-20220419-01 35522857 PMC9126056

[9] Lo C . The dengue vaccine dilemma. *Pharmaceutical Technology* . August 1, 2022. Accessed August 20, 2022. https://www.pharmaceutical‐technology.com/analysis/dangvaxia‐philippines/

[10] Tomacruz S . A year after Dengvaxia: immunization drops, measles outbreaks soar. *Rappler*. December 1, 2018. Accessed August 24, 2022. https://www.rappler.com/newsbreak/in‐depth/217912‐dengvaxia‐one‐year‐after‐outbreaks‐series‐part‐1/

[11] DOH . DOH, NTF: private sector allowed to procure COVID‐19 vaccines via tripartite agreements. March 22, 2021. Accessed October 25, 2022. https://doh.gov.ph/doh‐press‐release/DOH‐NTF‐PRIVATE‐SECTOR‐ALLOWED‐TO‐PROCURE‐COVID‐19‐VACCINES‐VIA‐TRIPARTITE‐AGREEMENTS

[12] World Bank . Transparency, good governance and anti‐corruption mechanisms. October 13, 2022. Accessed October 25, 2022. https://ppp.worldbank.org/public‐private‐partnership/overview/practical‐tools/good‐governance‐anticorruption

